# Supplementary material for: Histone functions as a cell-surface receptor for AGEs
Source: Nat Commun. 2022 May 27;13:2974. doi: 10.1038/s41467-022-30626-8 (PMC9142594; doi:10.1038/s41467-022-30626-8)
Supplement: Supplementary file 1 — Supplementary Information [file 41467_2022_30626_MOESM1_ESM.pdf]

Supplementary Information

Histone functions as a cell-surface receptor for AGEs

Masanori Itakura, Kosuke Yamaguchi, Roma Kitazawa, Sei-Young Lim, Yusuke Anan, Jun Yoshitake, Takahiro Shibata, Lumi Negishi, Hikari Sugawa, Ryoji Nagai, and Koji Uchida

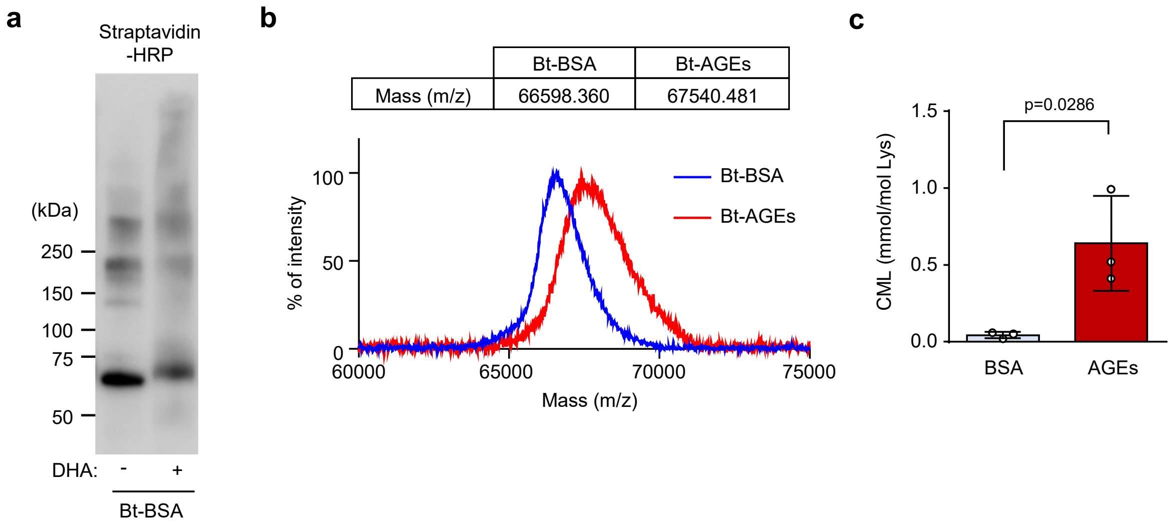

Figure S1. DHA modification of BSA

a, Bt-BSA (1.0 mg/ml) was incubated without or with 25 mM DHA for 72 h and dialyzed against PBS. The samples were denatured, separated by SDS-PAGE, transferred to PVDF membranes, and visualized using HRP- conjugated streptavidin. Representative image of three independent experiments. **b**, Bt-BSA incubated without or with DHA (Bt-AGEs) was analyzed by MALDI-TOF-MS. **c**, The CML contents in BSA or AGEs were measured by LC-MS/MS and normalized to the lysine content. The data were expressed as mmol/mol lysine. Data are mean ± S.D. (n=3, independent experiments). two-sided Student's *t* test.

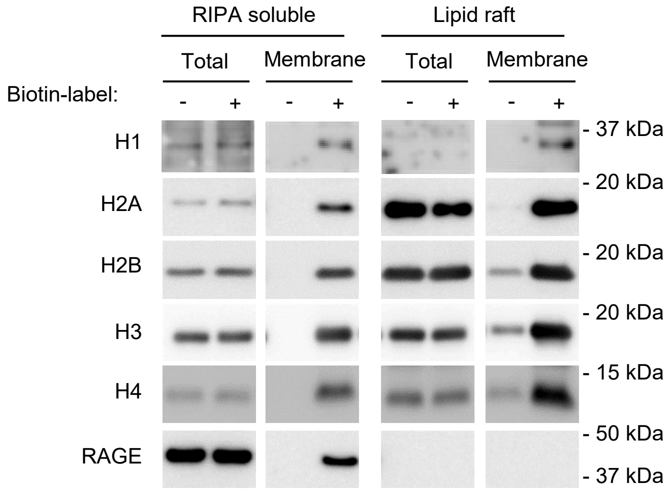

Figure S2. Membrane localization of histone proteins and RAGE

Splenocytes were labeled with membrane impermeable EZ-Link Sulfo-NHS-LC-Biotin for 30 min on ice and lysed in either RIPA soluble or lipid raft fraction. The cell lysates (Total) were subjected to pull-down with streptavidin-coupled magnetic beads, and the resulting precipitates (Membrane) were subjected to western blotting. Representative image of two independent experiments.

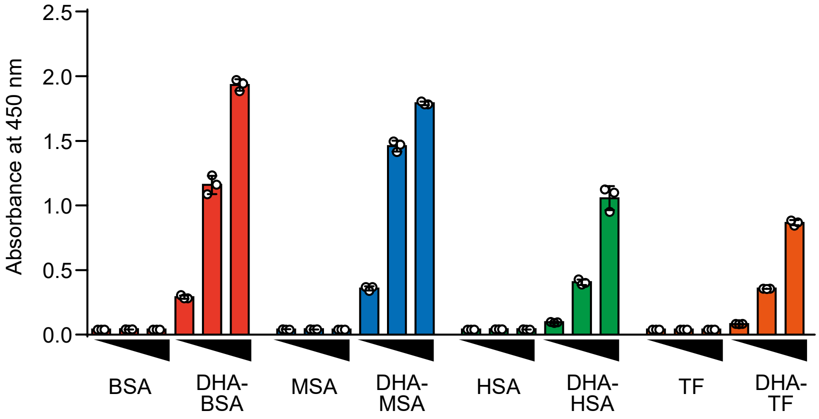

Figure S3. Histone binding activity was induced by DHA modification regardless of the type of protein

Binding of DHA-modified proteins to the recombinant histone H2B. Biotinylated BSA, mouse serum albumin (MSA), human serum albumin (HSA) and transferrin (TF) were modified with DHA. Unmodified and DHA-modified proteins (1, 3, 10 µg/ml) were incubated with recombinant histone H2B immobilized on ELISA plate. The binding to H2B was detected using streptavidin-HRP and color development (absorbance at 450 nm). Data are mean ± S.D. of triplicate samples and are representative of three independent experiments.

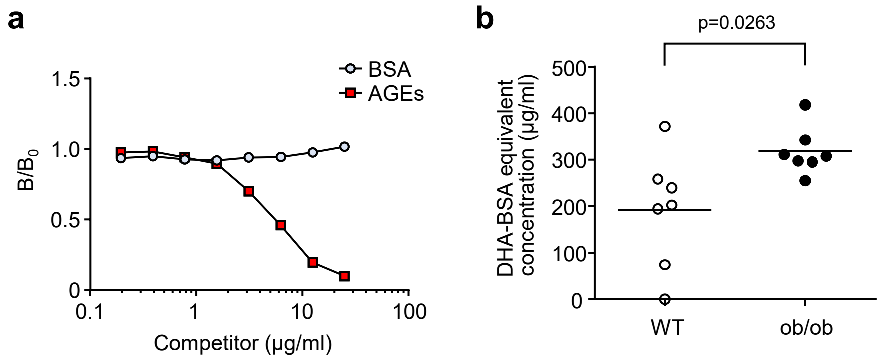

Figure S4. Detection of H2B binding molecules in sera from WT and ob/ob mice

a, Standard curves for the competitive solid-phase binding assay using BSA or AGEs as a competitor. BSA or AGEs were preincubated at indicated concentrations for 30 min on H2B coated plates before the addition of Bt- AGEs (1 μg/ml). **b**, Diluted sera (1:100) from 12 months old WT mice or ob/ob mice were used as competitors for the competitive solid-phase binding assay. The DHA-BSA equivalent concentrations were determined by fitting the sample signal values on the standard curve with the 4-parameter logistic equation. Data are mean ± S.D. (n=7, biologically independent experiments). two-sided Student's t test.

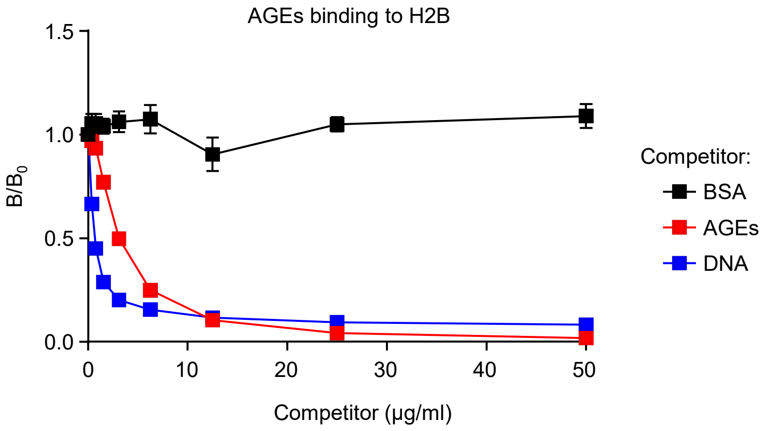

Figure S5. DNA inhibits A GEs binding to H2B

BSA, AGEs, or DNA were preincubated at indicated concentrations for 30 min on H2B coated plates before the addition of Bt-AGEs (1 µg/ml). The binding was detected using streptavidin-HRP. Data are mean ± S.D. of triplicate samples and are representative of three independent experiments.

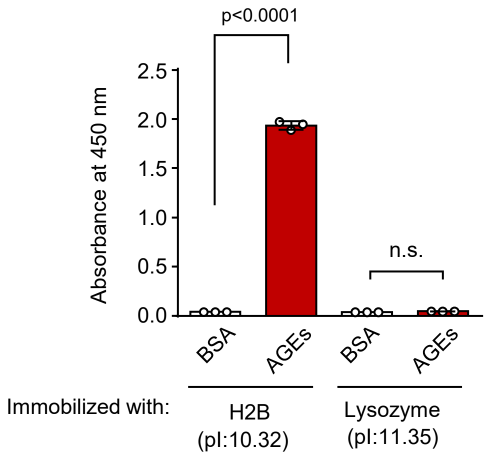

Figure S6. The binding of AGEs to H2B and lysozyme

H2B or lysozyme was immobilized on ELISA plate and incubated with Bt-BSA or Bt-AGEs (5 µg/ml). The binding was detected using streptavidin-HRP. Data are mean ± S.D. of triplicate samples and are representative of three independent experiments. Tukey-Kramer tests (two-sided), n.s., not significant ($p>0.05$).

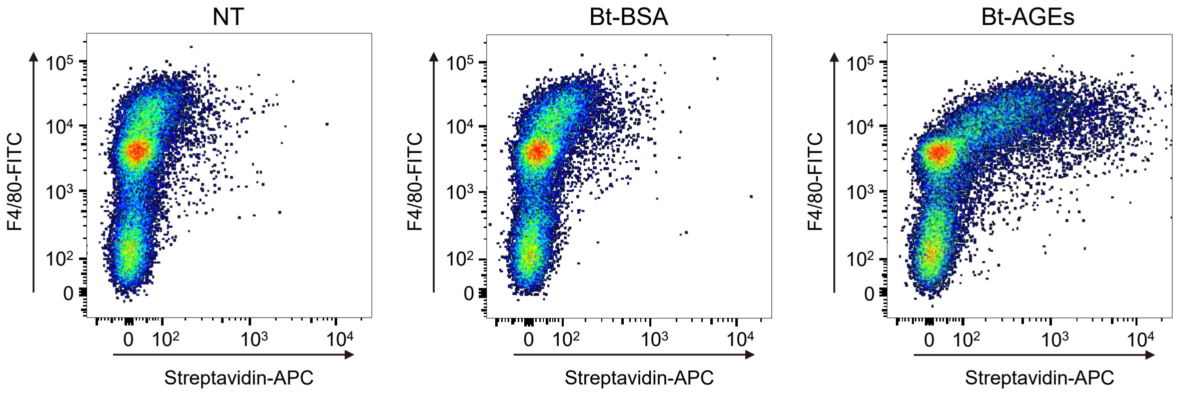

Figure S7. Binding of AGEs to mouse peritoneal macrophages

Peritoneal cells were incubated alone (NT) or with either Bt-BSA or Bt-AGEs at 4ºC for 15 min, stained with streptavidin-APC, CD11b, and F4/80 and analyzed by flow cytometry. A typical scatter plots of fluorescence intensities of F4/80-FITC vs streptavidin-APC were shown.

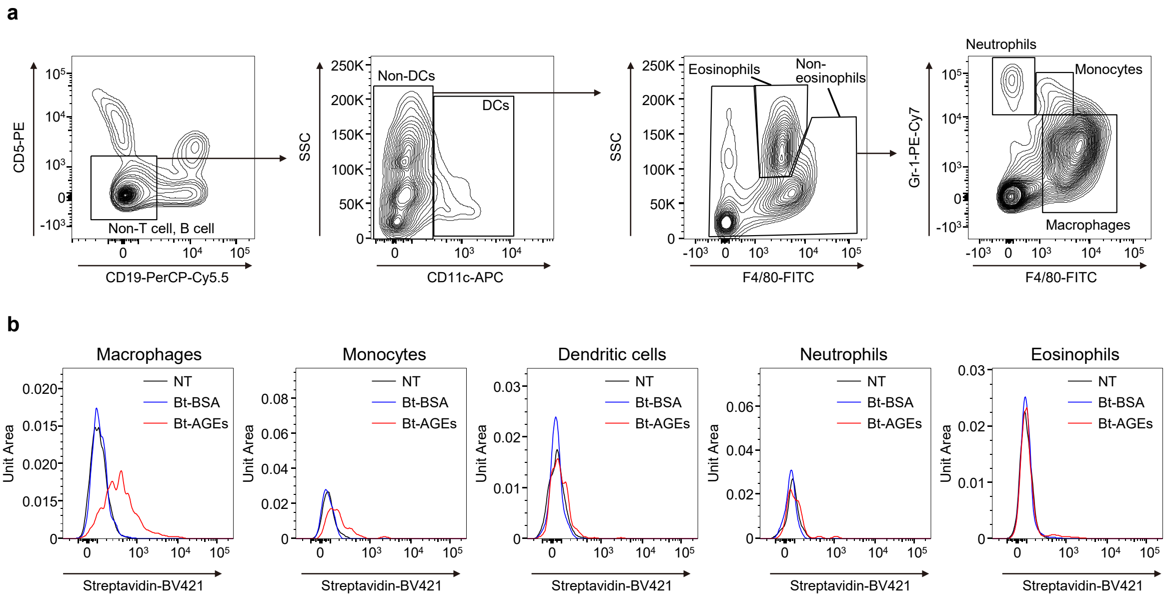

Figure S8. Cell-type specificity for AGEs binding

Peritoneal cells were incubated alone (NT) or with either Bt-BSA or Bt-AGEs at 4ºC for 15 min, stained with fluorochrome-conjugated antibodies and streptavidin-BV421, and analyzed by flow cytometry. **a**, Gating strategy for Macrophages (F4/80hi), Monocytes (Gr-1+, F4/80+), Dendritic cells (CD11c+), Neutrophils (Gr-1hi, F4/80-), Eosinophils (SSChi). **b**, The bindings of Bt-BSA or Bt-AGEs to Macrophages, Monocytes, Dendritic cells, Neutrophils, and Eosinophils were represented as histograms of fluorescent intensity of BV421.

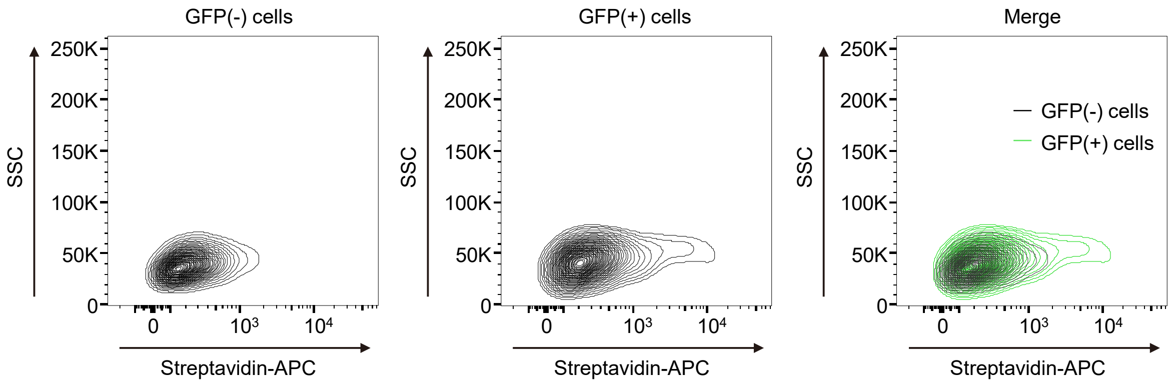

Figure S9. Effect of H2B overexpression on AGEs binding

J774A.1 cells were transiently transfected with a bicistronic construct encoding H2B followed by an IRES driving the translation of GFP. The cells were incubated with Bt-AGEs at 4ºC for 15 min, stained with streptavidin-APC, and analyzed by flow cytometry. GFP(-) cells and GFP (+) cells were gated, and the fluorescent intensities of APC, indicating the bindings of AGEs to cells, were represented in contour plots.

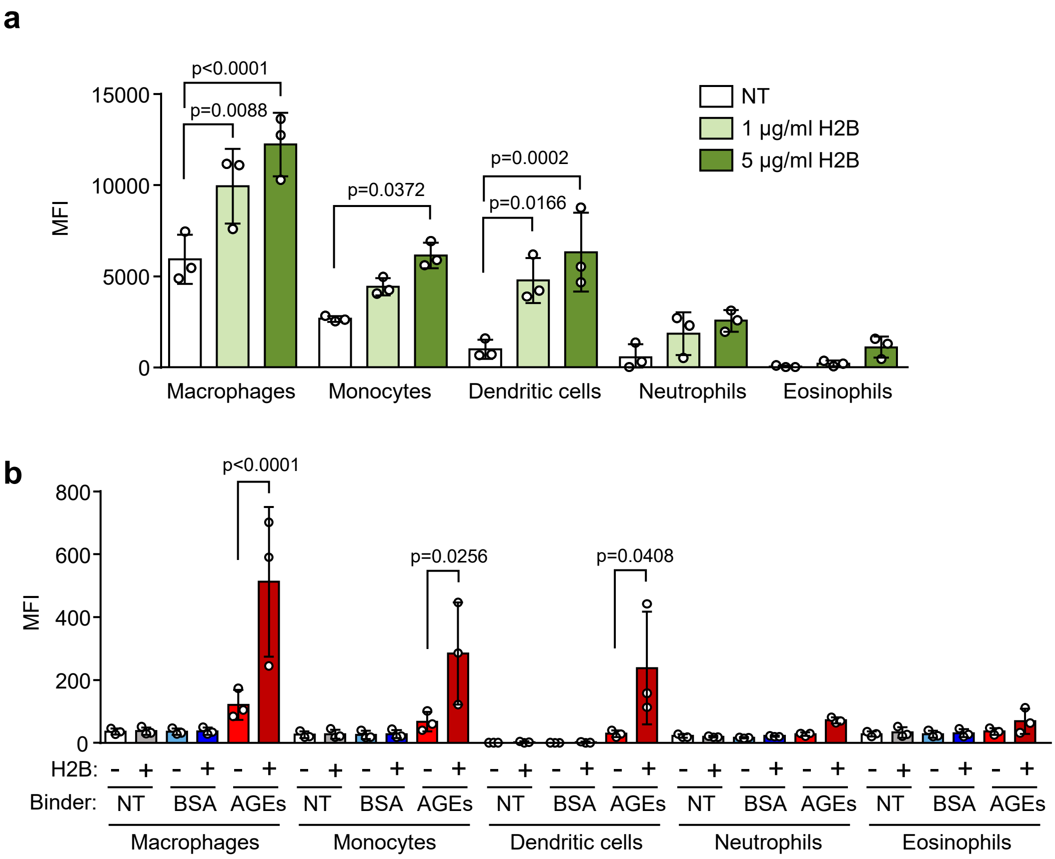

Figure S10 Supplementation of recombinant H2B enhances the binding of AGEs to macrophages, monocytes, and dendritic cells

a, Association of H2B to the c ell surface of macrophages, monocytes, and dendritic cells. Peritoneal cells were incubated with recombinant His-tagged histone H2B (1 or 5 μg/ml) at 4ºC for 15 min. After washing, cells were further incubated with biotin-conjugated anti-His-tag antibody, stained with fluorochrome-conjugated antibodies and streptavidin-BV421, and analyzed by flow cytometry. Macrophages, Monocytes, Dendritic cells, Neutrophils, and Eosinophils were gated as indicated in Fig. S8, and the bindings of recombinant H2B were expressed as median fluorescent intensity (MFI) of BV421. Data are mean ± S.D. (n=3, biologically independent experiments). Tukey-Kramer tests (two-sided). **b**, Peritoneal cells were incubated with 5 μg/ml recombinant H2B at 4ºC for 15 min. After washing, cells were incubated alone (NT) or with either Bt-BSA (BSA) or Bt-AGEs (AGEs) at 4ºC for 15 min, stained with fluorochrome-conjugated antibodies and streptavidin-BV421, and analyzed by flow cytometry. The bindings of Bt-BSA or Bt-AGEs were expressed as MFI of BV421. Data are mean ± S.D. (n=3, biologically independent experiments). Tukey-Kramer tests (two-sided).

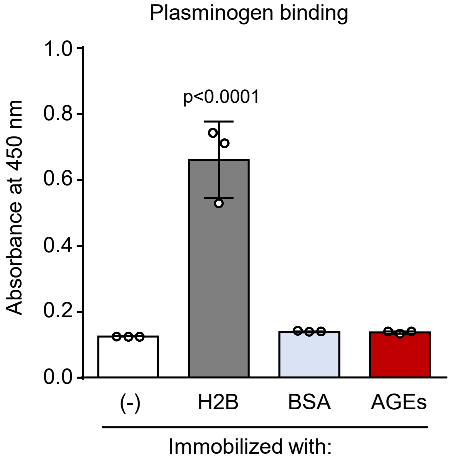

Figure S11. The binding of Plg to H2B, BSA, and AGEs

H2B, BSA, or AGEs were immobilized on ELISA plate and incubated with Bt-Plg (1 μg/ml). The binding was detected using streptavidin-HRP. Data are mean ± S.D. of triplicate samples and are representative of three independent experiments. Dunnett's test (two-sided), relative to the control.

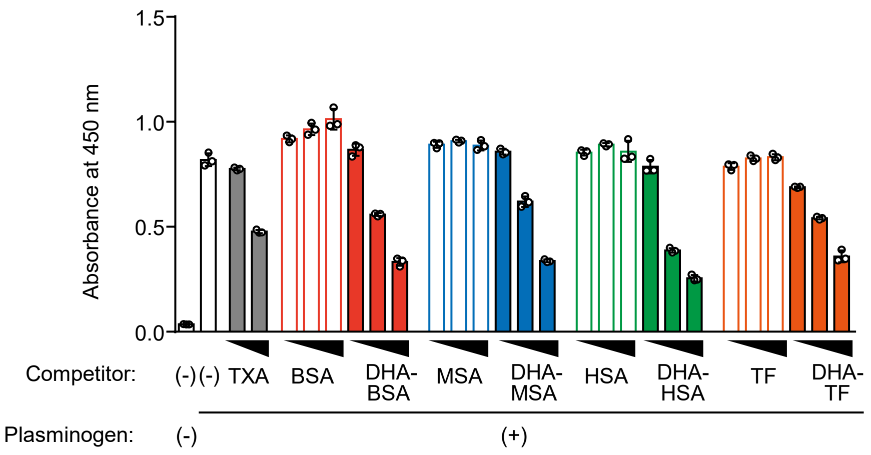

Figure S12. The inhibitory effect of AGEs on Plg binding to histone H2B is not dependent on the type of protein modified with DHA

Effect of DHA modification of BSA, MSA, HSA, and TF on Plg binding to histone H2B in vitro. Recombinant H2B coated on ELISA plate was preincubated with tranexamic acid (TXA, 1 nM and 1 μM), unmodified or DHA-modified proteins (1, 10, and 100 μg/ml) for 30 min, followed by treatment with Bt-Plg (1 μg/ml) for 60 min. Plg binding to H2B was detected with streptavidin-HRP and color development (absorbance at 450 nm). Data are mean ± S.D. of triplicate samples and are representative of three independent experiments.

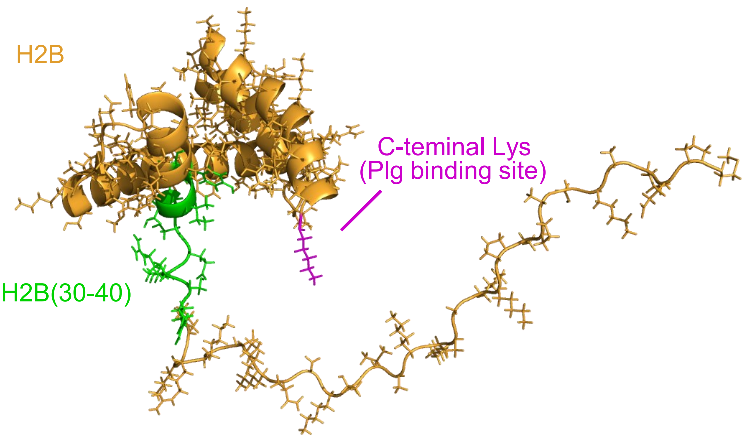

Figure S13. The putative AGEs binding site of H2B responsible for the inhibition of Plg binding

Structure of human histone H2B (PDB code: 2RVQ) is shown (orange). Plg binding site (carboxyl-terminal lysine) and AGEs binding region responsible for the inhibition of H2B-Plg binding (amino acid residues 30-40 of H2B) are shown in magenta and green, respectively.

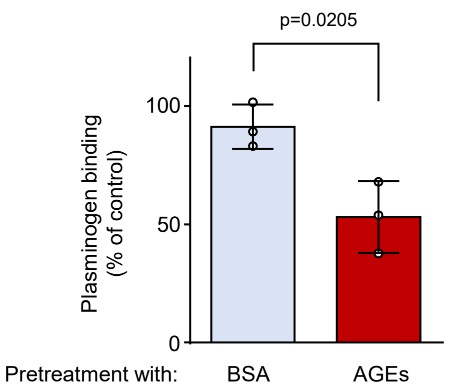

Figure S14. Pretreatment with AGEs is sufficient for the inhibition of Plg binding to macrophages

J774A.1 cells were preincubated with either 100 μg/ml BSA or AGEs at 4ºC for 15 min and then washed with HBSS-BSA. Cells w ere incubated with Bt-Plg for 15 min (in the absence or presence of 1 mM tranexamic acid), and Plg binding to cells was analyzed by FACS with streptavidin-APC. Plg binding (% of control) was calculated as described in the *Materials and Methods* section. Data are mean ± S.D. (n=3, biologically independent experiments). Two-sided Student's *t* test.

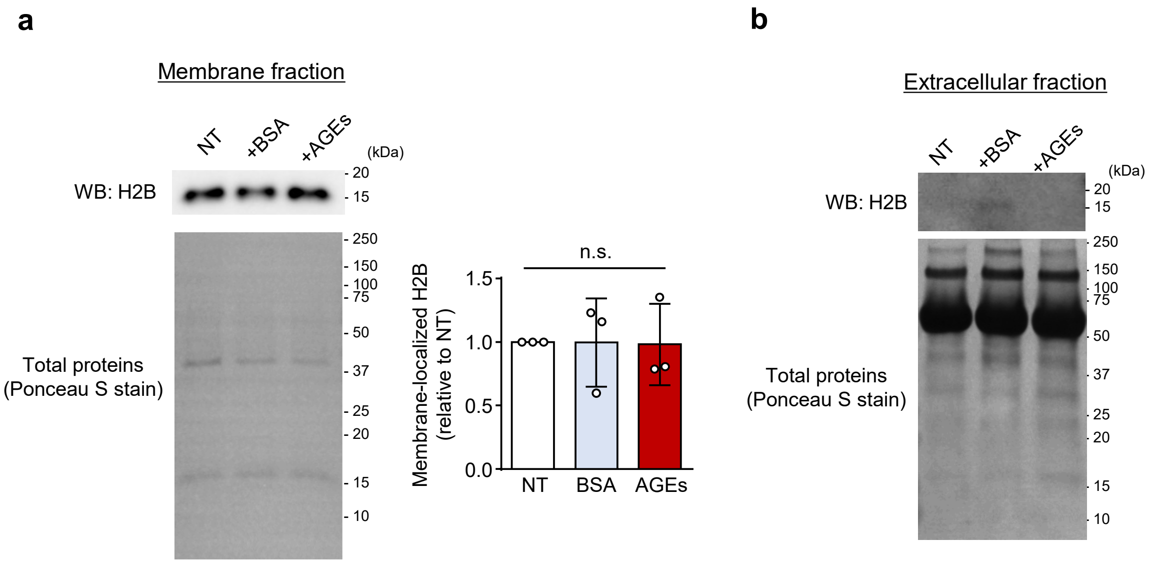

Figure S15. AGEs treatment does not cause H2B release from cell surface

J774A.1 cells were labeled with membrane impermeable EZ-Link Sulfo-NHS-LC-Biotin for 30 min on ice and then treated with eith er 100 µg/ml BSA or AGEs. After incubation for 15 min, cells were centrifuged, and supernatants were collected as “Extracellular fraction”. The cell lysates were subjected to pull-down with streptavidin-coupled magnetic beads, and the resulting precipitates were collected as “Membrane fraction”. Proteins in Membrane fraction (**a**) and Extracellular fraction (**b**) were transferred to PVDF membrane, stained with Ponceau S to visualize total proteins, and subjected to western blotting for H2B. Representative images of three independent experiments. Data are mean ± S.D. (n=3, biologically independent experiments). n.s., not significant (two-sided $p>0.05$), Tukey-Kramer tests.

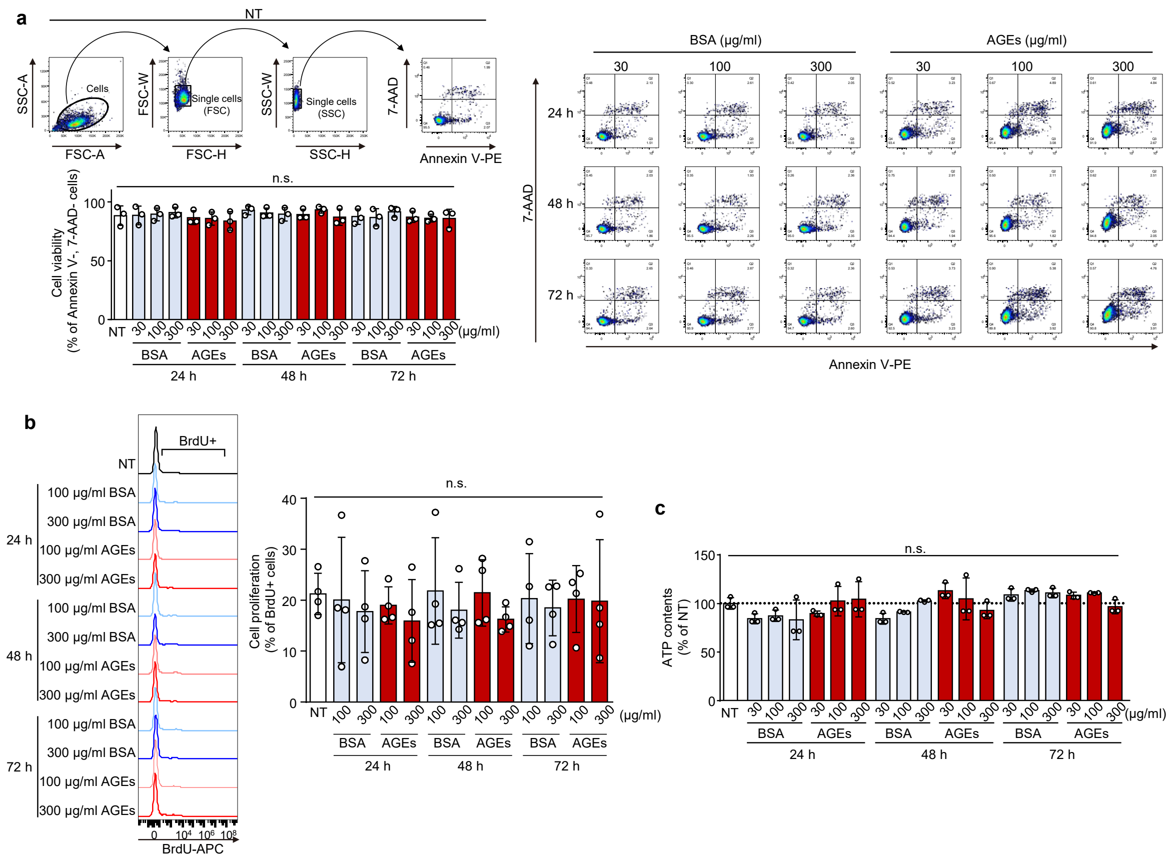

Figure S16. *In vitro* evaluation of cytotoxic effects and proliferative changes induced by BSA or AGEs

a, *In vitro* cell viability assay. J774A.1 cells were cultured in the presence of BSA or AGEs (30, 100, or 300 μg/ml) for the indicated time periods and stained with 7-AAD and PE-labeled Annexin V. The percentages of viable cells (Annexin V -, 7-AAD-) were calculated, and values were represented as mean ± S.D. (n=3, biologically independent experiments). n.s., not significant (two-sided p>0.05), Tukey-Kramer tests. **b**, BrdU incorporation assay. J774A.1 cells were cultured in the presence of BSA or AGEs (100 or 300 μg/ml) for the indicated time periods and labeled with BrdU for 45 min before being harvested. Cells were fixed and stained with APC-labeled anti-BrdU antibody, and detected by flow cytometry. The percentages of BrdU+ cells were calculated and values were represented as mean ± S.D. (n=4, biologically independent experiments). n.s., not significant (two-sided p>0.05), Tukey-Kramer tests. **c**, Measurement of ATP contents. J774A.1 cells were seeded in 96-well plate and then treated with either BSA or AGEs at indicated concentrations and time periods. After incubation, ATP contents were determined as described in the *Materials and Methods*. Data are mean ± S.D. (n=3, biologically independent experiments). n.s., not significant (two-sided p>0.05), Tukey-Kramer tests.

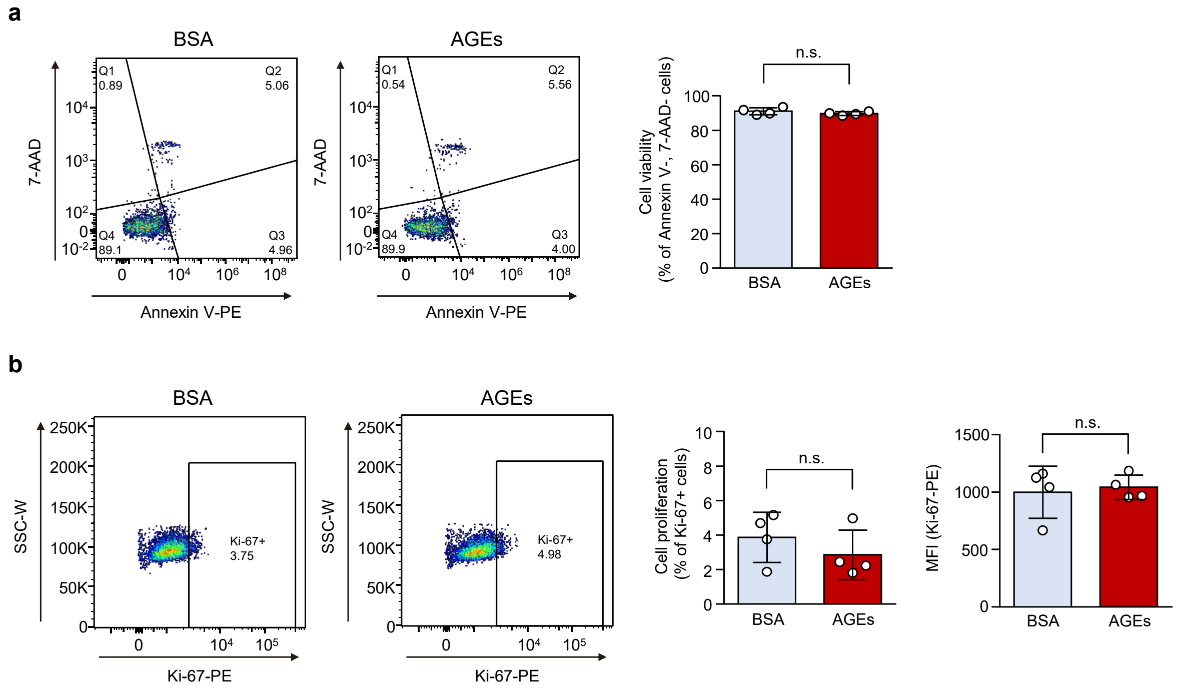

Figure S17 *In vivo* evaluation of cytotoxic effects and proliferative changes induced by BSA or AGEs treatment

a, *In vivo* cell viability assay. Mice were daily administered with BSA or AGEs (10 mg/kg/day) starting 24 h before TG injection and continuing until the mice were sacrificed. Peritoneal cells were collected 72 h after TG injection and stained with fluorochrome-conjugated antibodies, 7-AAD, and PE-labeled Annexin V. Macrophages were gated as indicated in Fig. S8. The percentages of viable cells (Annexin V -, 7-AAD-) were calculated and values were represented as mean ± S.D. (n=4, biologically independent experiments). n.s., not significant (two-sided $p>0.05$), Student's *t* test. **b**, *In vivo* proliferation assay. Peritoneal cells were collected 72 h after TG injection and stained with fluorochrome-conjugated antibodies. After fixation and permeabilization, the cells were stained with PE-labeled anti-Ki-67 antibody. Macrophages were gated, and both the percentages of proliferative cells (Ki-67+) and MFI of PE-Ki-67 were represented as mean ± S.D. (n=4, biologically independent experiments), respectively. n.s., not significant (two-sided $p>0.05$), Student's *t* test.

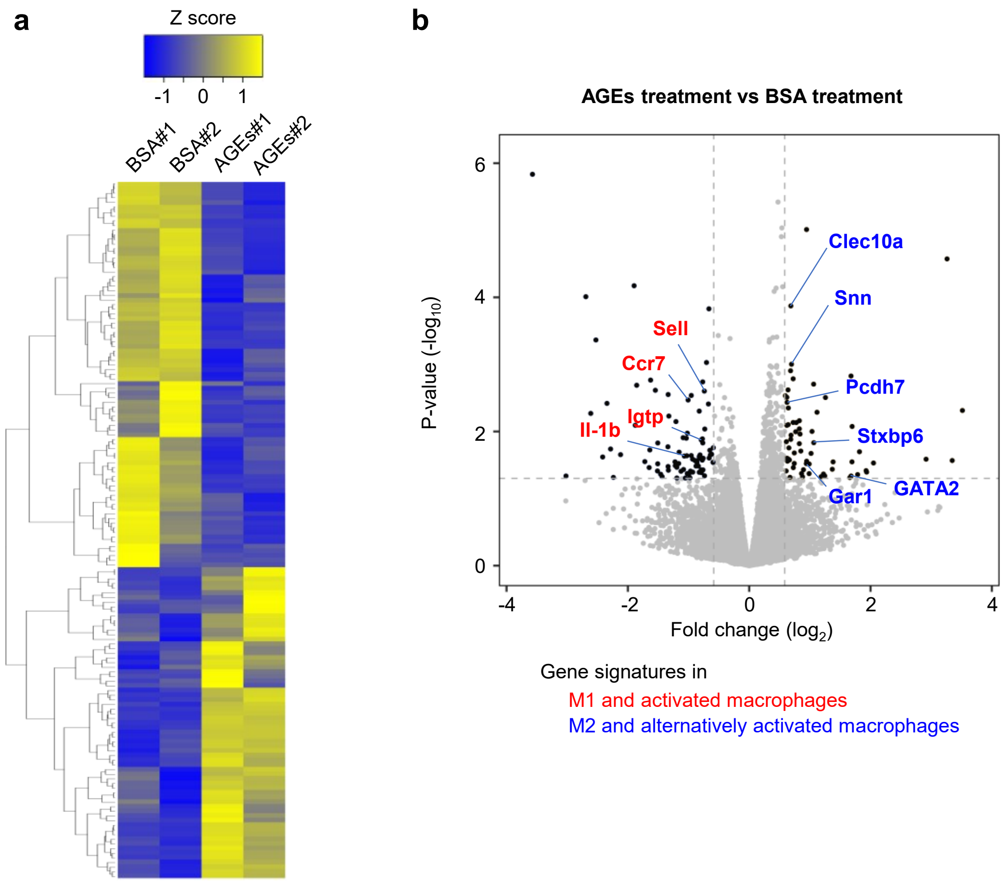

Figure S18. RNA-Seq analysis of isolated macrophages from the BSA- or AGEs-treated mice

Mice were daily administered with BSA or AGEs (10 mg/kg/day) starting 24 h before TG injection and continuing until the mice were sacrificed. Peritoneal macrophages were isolated from mice 72 h after intraperitoneal TG injection, and total RNA was subjected to RNA-seq analysis. **a**, Hierarchical clustering analysis of significant differentially expressed genes were generated using DESeq2. Data are expressed as z-score. **b**, Volcano plots showing fold-change and p-value for the comparisons of peritoneal macrophages isolated from AGEs-treated mice versus those from BSA-treated mice. Differentially expressed genes (|Fold change|>1.5 and p-value<0.05) are represented in black dots. Signature genes of "M1 and activated macrophages" and "M2 and alternatively activated macrophages" are shown in red and blue, respectively. Statistical significance of the differential expression data was determined using two-sided Wald test and adjustment using the Benjamini-Hochberg algorithm in DESeq2.

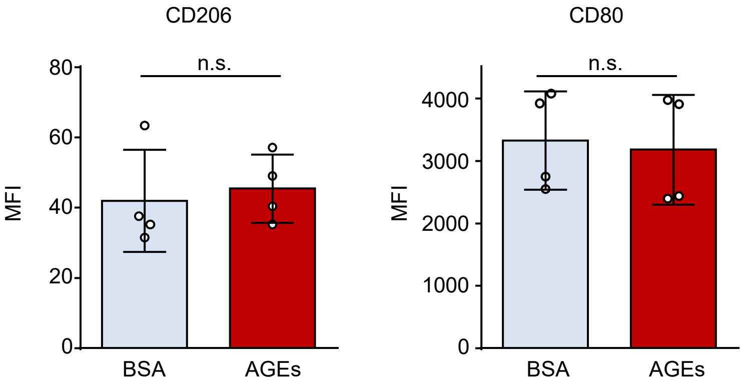

Figure S19. The effect of AGEs on the expression of protein marker for M1 and M2 peritoneal macrophages

Mice were daily administered with BSA or AGEs (10 mg/kg/day) starting 24 h before TG injection and continuing until the mice were sacrificed. Peritoneal cells were collected 72 h after TG injection, stained with either anti-CD80 (for M1 macrophages) or anti-CD206 (for M2 macrophages), and analyzed by FACS. Data are represented as mean fluorescent intensity (MFI) ± S.D. (n=4, biologically independent experiments). n.s., not significant (two-sided $p>0.05$), Student's *t* test.

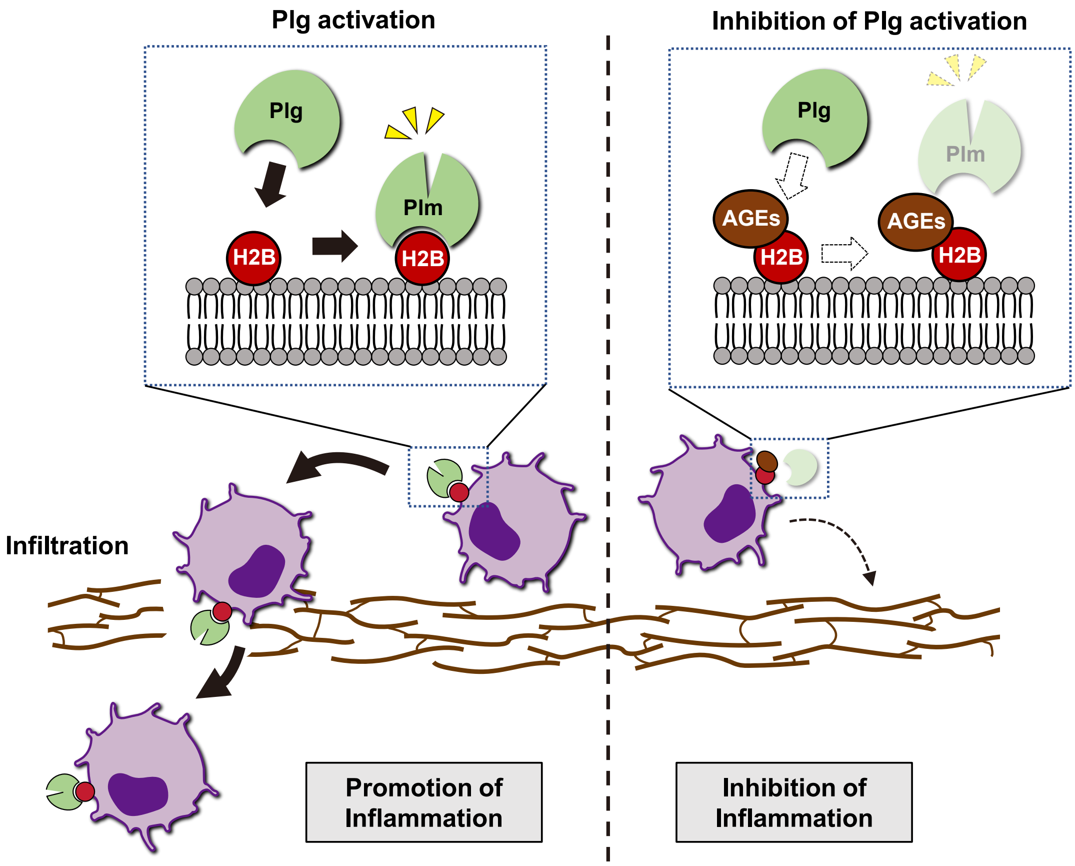

Figure S20. The graphical summary of the function of histone as a functional AGEs receptor

The interaction of plasminogen (Plg) with cell-surface Plg receptors, including histone H2B, accelerates conversion of Plg to plasmin (Plm) and enhances the catalytic activity of Plm. Plm formed on the cell surface is retained on the cell membrane and accelerates the infiltration of monocytes/macrophages (*left*). The binding of AGEs to the membrane-associated histone H2B resulted in suppression of Plg binding and subsequent inhibition of monocytes/macrophage recruitment to the site of inflammation (*right*).

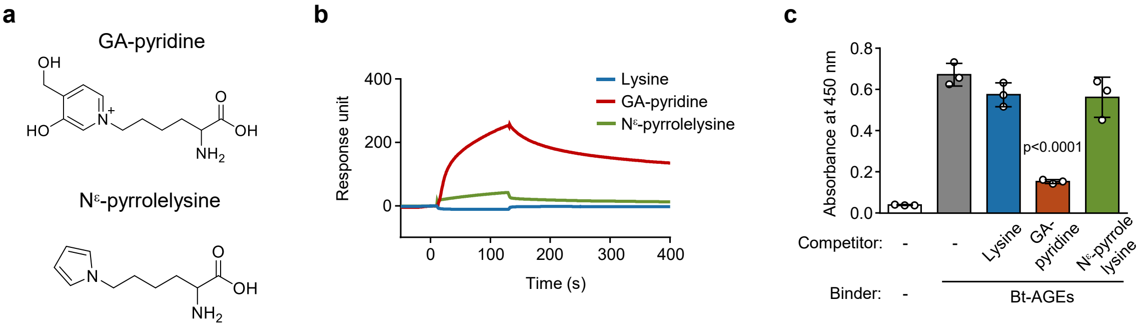

Figure S21. The binding of glycolaldehyde-lysine adducts to H2B

a, Chemical structures of glycolaldehyde-lysine adducts, GA-pyridine (upper) and N^{ε}-pyrrolelysine (lower). **b**, Surface plasmon resonance measurements. The interaction between H2B immobilized on a Biacore sensor chip NTA and unmodified lysine, GA-pyridine, or N^{ε}-pyrrolelysine (250 μM) was monitored. **c**, Effect of unmodified lysine, GA-pyridine, or N^{ε}-pyrrolelysine on the binding of AGEs to H2B. Recombinant H2B coated on ELISA plate was preincubated with either unmodified lysine, GA-pyridine, or N^{ε}-pyrrolelysine (250 μM) for 30 min, followed by treatment with Bt-AGEs (5 μg/ml) for 60 min. The binding of AGEs to H2B was detected with streptavidin-HRP and color development (absorbance at 450 nm). Data are mean ± S.D. of triplicate samples and are representative of three independent experiments. Dunnett's test (two-sided), relative to the treatment with Bt-AGEs alone.

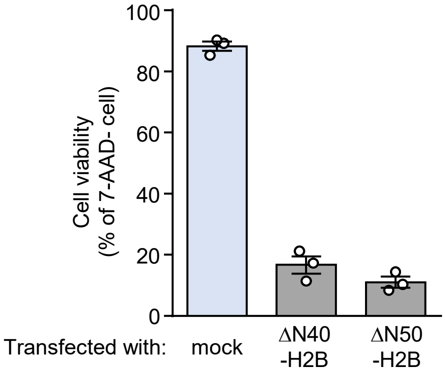

Figure S22. The marked cell death induced by the expression of truncated H2B

Effect of truncated H2B overexpression on cell viability. J774A.1 cells were transiently transfected with a construct encoding deletion mutants of H2B (ΔN40 and ΔN50). After 24-hours incubation, the cells were stained with 7-AAD and analyzed by flow cytometry. The percentages of viable cells (7-AAD-) were calculated, and values were represented as mean ± S.D. (n=3, biologically independent experiments).

Table S1. Identification of histone H2B as an AGEs-binding protein

Band	Accession	Description	Coverage [%]	Score Sequest HT	# Missed Cleavages	Charge	m/z [Da]	XCorr	Annotated Sequence
10 kDa protein	B2RTM0	Histone H4	65.04854	35.03	2	2	422.2624	1.67	[K].GLGKGGAKR.[H]
					0	2	663.8534	2.03	[K].TVTAmDVVYALK.[R]
					1	3	526.635	1.38	[R].ISGLIYEETRGVLK.[V]
					1	3	489.606	1.65	[K].TVTAMDVVYALKR.[Q]
					2	3	537.6361	1.72	[R].KTVTAmDVVYALKR.[Q]
					0	2	567.7769	1.82	[R].DAVTYTEHAK.[R]
					0	2	567.7765	2.47	[R].DAVTYTEHAK.[R]
					0	2	567.775	2.57	[R].DAVTYTEHAK.[R]
					0	2	567.7744	1.3	[R].DAVTYTEHAK.[R]
					0	2	663.3827	1.74	[R].DNIQGITKPAIR.[R]
					0	2	663.3835	2.17	[R].DNIQGITKPAIR.[R]
					0	2	495.2934	2.24	[K].VFLENVIR.[D]
					0	2	663.3821	1.86	[R].DNIQGITKPAIR.[R]
					0	2	663.3826	2.25	[R].DNIQGITKPAIR.[R]
					0	2	590.8168	3.17	[R].ISGLIYEETR.[G]
					0	2	590.8154	3.3	[R].ISGLIYEETR.[G]
					2	3	537.6367	2.38	[R].KTVTAmDVVYALKR.[Q]
					0	2	663.3819	2.87	[R].DNIQGITKPAIR.[R]
					0	2	655.8548	2.49	[K].TVTAMDVVYALK.[R]

Band	Accession	Description	Coverage [%]	Score Sequest HT	# Missed Cleavages	Charge	m/z [Da]	XCorr	Annotated Sequence
15 kDa protein	Q64426	Histone H2A (Fragment)	30.65693	30.61	0	3	644.394	4.2	[KR].VTIAQGGVLPNIQAVLLPK.[K]
					0	3	644.3939	3.07	[KR].VTIAQGGVLPNIQAVLLPK.[K]
					0	2	472.7692	2.13	[R].AGLQFPVGR.[VI]
					0	2	472.77	2.95	[R].AGLQFPVGR.[VI]
					0	2	431.2021	1.6	[R].NDEELNK.[L]
					0	2	425.7665	2.05	[R].HLQLAIR.[NG]
					0	2	425.7676	2.03	[R].HLQLAIR.[NG]
					0	2	425.7661	1.62	[R].HLQLAIR.[NG]
					1	2	846.9569	2.42	[R].HLQLAIRNDEELNK.[L]
					0	2	472.7702	2.86	[R].AGLQFPVGR.[VI]
					0	2	472.7699	2.61	[R].AGLQFPVGR.[VI]
					0	2	472.7703	2.29	[R].AGLQFPVGR.[VI]
					0	3	644.3936	2.38	[KR].VTIAQGGVLPNIQAVLLPK.[K]

Band	Accession	Description	Coverage [%]	Score Sequest HT	# Missed Cleavages	Charge	m/z [Da]	XCorr	Annotated Sequence
15 kDa protein	B2RTK3	Histone H2B	14.2857142857143	4.52	0	2	352.1913	2.12	[R].IAGEASR.[L]
					0	3	390.2044	2.39	[K].QVHPDTGISSK.[A]

Band	Accession	Description	Coverage [%]	Score Sequest HT	# Missed Cleavages	Charge	m/z [Da]	XCorr	Annotated Sequence
25 kDa protein	Q5SZA3	Histone cluster 1, H1c	28.77358	61.7	1	3	352.5592	1.75	[K].AKKPAAAAVTK.[K]
					0	2	599.84	2.62	[K].ASGPPVSELITK.[A]
					0	2	599.8392	2.11	[K].ASGPPVSELITK.[A]
					0	2	554.2894	2.56	[K].ALAAAGYDVEK.[N]
					1	2	789.8984	2.37	[K].ALAAAGYDVEKNNSR.[I]
					1	2	789.8997	2.46	[K].ALAAAGYDVEKNNSR.[I]
					1	3	526.9365	1.54	[K].ALAAAGYDVEKNNSR.[I]
					0	2	554.2902	2.62	[K].ALAAAGYDVEK.[N]
					0	2	554.2883	2.29	[K].ALAAAGYDVEK.[N]
					1	3	442.9272	3.57	[R].KASGPPVSELITK.[A]
					0	2	554.2896	2.25	[K].ALAAAGYDVEK.[N]
					1	2	487.3068	3.16	[R].SGVSLAALKK.[AS]
					1	2	789.902	2.67	[K].ALAAAGYDVEKNNSR.[I]
					1	2	487.3068	1.65	[R].SGVSLAALKK.[AS]
					1	2	663.8862	1.99	[R].KASGPPVSELITK.[A]
					1	2	663.8873	2.38	[R].KASGPPVSELITK.[A]
					1	3	352.5586	2.21	[K].AKKPAAAAVTK.[K]
					1	3	442.9274	2.83	[R].KASGPPVSELITK.[A]
					1	3	352.5588	2.2	[K].AKKPAAAAVTK.[K]
					1	2	663.8856	2.8	[R].KASGPPVSELITK.[A]
					1	2	663.8868	1.99	[R].KASGPPVSELITK.[A]
					0	2	428.7681	1.82	[K].KPAAAAVTK.[K]
					1	2	583.8149	1.88	[K].GTGASGSFKLNK.[K]
					1	3	442.9269	2.85	[R].KASGPPVSELITK.[A]
					1	3	526.9363	1.53	[K].ALAAAGYDVEKNNSR.[I]
					0	2	599.84	1.94	[K].ASGPPVSELITK.[A]
					0	2	599.8391	1.29	[K].ASGPPVSELITK.[A]
					1	2	663.8862	2.04	[R].KASGPPVSELITK.[A]
					0	2	428.7679	1.79	[K].KPAAAAVTK.[K]
					1	3	442.9278	2.66	[R].KASGPPVSELITK.[A]

Table S2. List of the histone H2B fragments

Peptide name	Amino acid sequence
H2B (FL)	MPEPALSAPAPKKGSKKAVTKAQKKDGKKRKRSRKESYSIYVYK VLKQVHPDTGISSKAMGIMNSFVNDIFERIAGEASRLAHYNKRST ITSREIQTAVRLLLPGELAKHAVSEGTKAVTKYTSAK
H2B (1-35)	MPEPALSAPAPKKGSKKAVTKAQKKDGKKRKRSRK
H2B (DN35)	ESYSIYVYKVLKQVHPDTGISSKAMGIMNSFVNDIFERIAGEASRL AHYNKRSTITSREIQTAVRLLLPGELAKHAVSEGTKAVTKYTSAK
H2B Scrambled 1	VKPKKGPMKKKGDTSRASAEPKKKPRAKSAQAKRK
H2B Scrambled 2	GKDATKRKSGKKAKEQSARASKKPKRPPKVPMAKK
H2B Scrambled 3	RAKAKKVPRSAMKKKPKSPGDKKKKQGASRATPKE

Table S3. Primer list

Name	Sense/Antisense	Sequence (5'-3')
IL-1β	Sense	TTGACGGACCCCAAAAGAT
	Antisense	AGCTGGATGCTCTCATCAGG
IL-6	Sense	CAACGATGATGCACTTGCAGA
	Antisense	CTCCAGGTAGCTATGGTACTCCAGA
Arg-1	Sense	AGACAGCAGAGGAGGTGAAGAGTAC
	Antisense	GGTAGTCAGTCCCTGGCTTATGGT
IL-10	Sense	GCCAGAGCCACATGCTCCTA
	Antisense	GATAAGGCTTGGCAACCCAAGTAA
CD206	Sense	TCTTTGCCTTTCCCAGTCTCC
	Antisense	TGACACCCAGCGGAATTTC
β-actin	Sense	CATCCGTAAAGACCTCTATGCCAAC
	Antisense	ATGGAGCCACCGATCCACA
