# Supplementary material for: Histone functions as a cell-surface receptor for AGEs
Source: Nat Commun. 2022 May 27;13:2974. doi: 10.1038/s41467-022-30626-8 (PMC9142594; doi:10.1038/s41467-022-30626-8)
Supplement: Supplementary file 3 — Reporting Summary [file 41467_2022_30626_MOESM3_ESM.pdf]

nature portfolio

Corresponding author(s): Koji Uchida

Last updated by author(s): Mar 31, 2022

Reporting Summary

Nature Portfolio wishes to improve the reproducibility of the work that we publish. This form provides structure for consistency and transparency in reporting. For further information on Nature Portfolio policies, see our Editorial Policies and the Editorial Policy Checklist.

Statistics

For all statistical analyses, confirm that the following items are present in the figure legend, table legend, main text, or Methods section.

n/a	Confirmed	
☐	☒	The exact sample size (*n*) for each experimental group/condition, given as a discrete number and unit of measurement
☐	☒	A statement on whether measurements were taken from distinct samples or whether the same sample was measured repeatedly
☐	☒	The statistical test(s) used AND whether they are one- or two-sided<br>*Only common tests should be described solely by name; describe more complex techniques in the Methods section.*
☒	☐	A description of all covariates tested
☒	☐	A description of any assumptions or corrections, such as tests of normality and adjustment for multiple comparisons
☐	☒	A full description of the statistical parameters including central tendency (e.g. means) or other basic estimates (e.g. regression coefficient) AND variation (e.g. standard deviation) or associated estimates of uncertainty (e.g. confidence intervals)
☐	☒	For null hypothesis testing, the test statistic (e.g. *F*, *t*, *r*) with confidence intervals, effect sizes, degrees of freedom and *P* value noted<br>*Give P values as exact values whenever suitable.*
☒	☐	For Bayesian analysis, information on the choice of priors and Markov chain Monte Carlo settings
☒	☐	For hierarchical and complex designs, identification of the appropriate level for tests and full reporting of outcomes
☒	☐	Estimates of effect sizes (e.g. Cohen's *d*, Pearson's *r*), indicating how they were calculated

Our web collection on statistics for biologists contains articles on many of the points above.

Software and code

Policy information about availability of computer code

Data collection	BD FACSSuite version 1.0.3.2942 and FACSDiva version 8.0 software (BD Pharmingen) for flow cytmetry. Olympus software FV10-ASW Version 04.02.03.06(Olympus) for confocal imaging. NovaSeq6000 instrument (Illumina) for RNA-seq analysis.
Data analysis	Data analysis was performed using GraphPad Prism 6.07 (statistic analysis), Image J Fiji version 1.52n (imaging analysis), FlowJo v10 software (flow cytmetric analysis), Olympus software FV10-ASW (confocal image analysis), HISAT2 version 2.1.0 (RNA-seq mapping to the reference genome), StringTie version 2.1.3b (FPKM and TPM caluclation), and DESeq2 (DEG determination).

For manuscripts utilizing custom algorithms or software that are central to the research but not yet described in published literature, software must be made available to editors and reviewers. We strongly encourage code deposition in a community repository (e.g. GitHub). See the Nature Portfolio guidelines for submitting code & software for further information.

Data

Policy information about availability of data

All manuscripts must include a data availability statement. This statement should provide the following information, where applicable:

- Accession codes, unique identifiers, or web links for publicly available datasets
- A description of any restrictions on data availability
- For clinical datasets or third party data, please ensure that the statement adheres to our policy

The RNA-Seq data have been deposited into the Gene Expression Omnibus (accession code GSE195558). The structure of histone H2B used in this study is available in the Protein Data Bank (PDB) under accession codes 2RVQ. The source data underlying for the main figures and supplementary figures are provided as a Source Data file,which is included in the submission.

Field-specific reporting

Please select the one below that is the best fit for your research. If you are not sure, read the appropriate sections before making your selection.

☒ Life sciences ☐ Behavioural & social sciences ☐ Ecological, evolutionary & environmental sciences

For a reference copy of the document with all sections, see nature.com/documents/nr-reporting-summary-flat.pdf

Life sciences study design

All studies must disclose on these points even when the disclosure is negative.

Sample size	We did not use statistical methods to determine sample size. We determined the sample size based on similar experimental setups in previous publications.
Data exclusions	No data were excluded, except for those from mice that died during the course of the experiment.
Replication	Biological replicates in each experiments are defined in the figure legends. In the result of solid-phase binding assay, samples were replicated in triplicates and data are representative of two or three independent experiment.
Randomization	All cell samples were randomly selected for experiments. The mice in each experiment were divided into various groups according to their ages and weights in random order.
Blinding	Investigators were not blinded during data acquisition. Measurements and data reported did not require subjective judgement or interpretation from the investigators.

Reporting for specific materials, systems and methods

We require information from authors about some types of materials, experimental systems and methods used in many studies. Here, indicate whether each material, system or method listed is relevant to your study. If you are not sure if a list item applies to your research, read the appropriate section before selecting a response.

Materials & experimental systems

n/a	Involved in the study	
☐	☒	Antibodies
☐	☒	Eukaryotic cell lines
☒	☐	Palaeontology and archaeology
☐	☒	Animals and other organisms
☒	☐	Human research participants
☒	☐	Clinical data
☒	☐	Dual use research of concern

Methods

n/a	Involved in the study	
☒	☐	ChIP-seq
☐	☒	Flow cytometry
☒	☐	MRI-based neuroimaging

Antibodies

Antibodies used

Rat anti-F4/80 (clone:BM8, BioLegend, #123108)
Rat anti-CD11b (clone:M1/70, BD Pharmingen, #553311)
Rat anti-Gr-1 (clone:RB6-8C5, BioLegend, #108416)
Hamster anti-CD11c (clone:HL3, BD Pharmingen, #550261)
Rat anti-CD5 (clone:53-7.3, BioLegend, #100626)
Rat anti-CD19 (clone:6D5, BioLegend, #115512)
Rat anti-CD16/CD32 (clone:93, BioLegend, #101302)
Mouse anti-Histone H2B (clone:5HH2-2A8, Millipore, #05-1352)
Mouse anti-Histone H1 (clone:H-2, Santa Cruz, sc-393358)
Mouse anti-Histone H2A (clone:938CT5.1.1, Santa Cruz, sc-517336)
Mouse anti-Histone H2B (clone:A-6, Santa Cruz, sc-515808)
Mouse anti-Histone H3 (clone:1G1, Santa Cruz, sc-517576)
Mouse anti-Histone H4 (clone:F-9, Santa Cruz, sc-25260)
Mouse anti-RAGE (clone:A-9, Santa Cruz, sc-365154)
Mouse anti-AGER1 (clone:E-9, Santa Cruz, sc-74408)
Mouse anti-CD36 (clone:SMϕ, Santa Cruz, sc-7309)
Rat anti-Ki-67 (clone:16A8, Biolegend, #652404)
Mouse anti-BrdU (clone:3D4, BD Pharmingen, #552598)
Rabbit anti-Histone H1.2 (Proteintech, 19649-1-AP)
Mouse histone H4 (clone:L64C1, Cell signaling, #2935)
Rabbit anti-MMP-9 (clone:AB19016, Millipore, #AB19016)

Goat anti-mouse IgG-Alexa Fluor 488 conjugate (Invitrogen, A11001)
Horse anti-mouse IgG-HRP conjugate (Cell signaling, #7076)
Goat anti-rabbit IgG-HRP conjugate (Cell signaling, #7074)

Validation

All antibodies were obtained from commercial sources and validated according to the manufacturer. We confirmed with negative and positive control before the study.
Abbreviation for species reactivity: H-human, M-mouse, R-rat, Mk-monkey
Abbreviation for application: WB-western blotting, IP-immunoprecipitation, IC-immunocytochemistry, IF-immunofluorescece, IH-immunohistochemistry(Tissue), IH(P)-immunohistochemistry(Paraffin), FC-flow cytmetry

Rat anti-F4/80 (clone:BM8, BioLegend, #123108)
Species reactivity: M
Application validated by manufacturer: FC
Validation statements on the manufacturer's website: Quality Tested
https://www.biolegend.com/ja-jp/products/fitc-anti-mouse-f4-80-antibody-4067?GroupID=BLG5319

Rat anti-CD11b (clone:M1/70, BD Pharmingen, #553311)
Species reactivity: H, M
Application validated by manufacturer: FC
Validation statements on the manufacturer's website: Routinely Tested
https://www.bdbiosciences.com/en-au/products/reagents/flow-cytometry-reagents/research-reagents/single-color-antibodies-ruo/pe-rat-anti-cd11b.553311

Rat anti-Gr-1 (clone:RB6-8C5, BioLegend, #108416)
Species reactivity: M
Application validated by manufacturer: FC
Validation statements on the manufacturer's website: Quality Tested
https://www.biolegend.com/ja-jp/products/pe-cyanine7-anti-mouse-ly-6g-ly-6c-gr-1-antibody-1931?GroupID=BLG4876

Hamster anti-CD11c (clone:HL3, BD Pharmingen, #550261)
Species reactivity: M
Application validated by manufacturer: FC
Validation statements on the manufacturer's website: Routinely Tested
https://www.bdbiosciences.com/en-au/products/reagents/flow-cytometry-reagents/research-reagents/single-color-antibodies-ruo/apc-hamster-anti-mouse-cd11c.550261

Rat anti-CD5 (clone:53-7.3, BioLegend, #100626)
Species reactivity: M
Application validated by manufacturer: FC
Validation statements on the manufacturer's website: Quality Tested
https://www.biolegend.com/ja-jp/products/apc-anti-mouse-cd5-antibody-9923?GroupID=BLG6762

Rat anti-CD19 (clone:6D5, BioLegend, #115512)
Species reactivity: M
Application validated by manufacturer: FC
Validation statements on the manufacturer's website: Quality Tested
https://www.biolegend.com/ja-jp/products/apc-anti-mouse-cd19-antibody-1526?GroupID=BLG10556

Rat anti-CD16/CD32 (clone:93, BioLegend, #101302)
Species reactivity: M
Application validated by manufacturer: FC
Validation statements on the manufacturer's website: Quality Tested
https://www.biolegend.com/ja-jp/search-results/purified-anti-mouse-cd16-32-antibody-190

Mouse anti-Histone H2B (clone:5HH2-2A8, Millipore, #05-1352)
Species reactivity: H, M, R
Application validated by manufacturer: WB, IC
Validation statements on the manufacturer's website: Anti-Histone H2B Antibody, clone 5HH2-2A8 is a Mouse Monoclonal Antibody for detection of Histone H2B also known as Histone H2B, histone 2 H2be & has been validated in WB, ICC
https://www.merckmillipore.com/JP/ja/product/Anti-Histone-H2B-Antibody-clone-5HH2-2A8,MM_NF-05-1352

Mouse anti-Histone H1 (clone:H-2, Santa Cruz, sc-393358)
Species reactivity: H, M, R
Application validated by manufacturer: IP, WB, IH(P), ELISA, IF, FC
https://datasheets.scbt.com/sc-393358.pdf

Mouse anti-Histone H2A (clone:938CT5.1.1, Santa Cruz, sc-517336)
Species reactivity: H
Application validated by manufacturer: WB, IP, IF, IH(P)
https://datasheets.scbt.com/sc-517336.pdf

Mouse anti-Histone H2B (clone:A-6, Santa Cruz, sc-515808)
Species reactivity: H, M, R
Application validated by manufacturer: WB, IP, IF, IH(P), ELISA
https://datasheets.scbt.com/sc-515808.pdf

Mouse anti-Histone H3 (clone:1G1, Santa Cruz, sc-517576)
Species reactivity: H, M, R
Application validated by manufacturer: WB, IF
https://datasheets.scbt.com/sc-517576.pdf

Mouse anti-Histone H4 (clone:F-9, Santa Cruz, sc-25260)
Species reactivity: H, M, R
Application validated by manufacturer: WB, IP, IF, ELISA
https://datasheets.scbt.com/sc-25260.pdf

Mouse anti-RAGE (clone:A-9, Santa Cruz, sc-365154)
Species reactivity: H, M, R
Application validated by manufacturer: WB, IP, IF, IH(P), ELISA
https://datasheets.scbt.com/sc-365154.pdf

Mouse anti-AGER1 (clone:E-9, Santa Cruz, sc-74408)
Species reactivity: H, M, R
Application validated by manufacturer: WB, IP, IF, IH(P), ELISA
https://datasheets.scbt.com/sc-74408.pdf

Mouse anti-CD36 (clone:SMϕ, Santa Cruz, sc-7309)
Species reactivity: H, M, R
Application validated by manufacturer: WB, IP, IF, IH(P), FC
https://datasheets.scbt.com/sc-7309.pdf

Rat anti-Ki-67 (clone:16A8, Biolegend, #652404)
Species reactivity: M
Application validated by manufacturer: IC, FC
Validation statements on the manufacturer's website: Quality Tested
https://www.biolegend.com/ja-jp/products/pe-anti-mouse-ki-67-antibody-8134

Mouse anti-BrdU (clone:3D4, BD Pharmingen, #552598)
Species reactivity: H, M, R
Application validated by manufacturer: FC, IF, IH
Validation statements on the manufacturer's website: Routinely Tested
https://www.bdbiosciences.com/en-us/products/reagents/flow-cytometry-reagents/research-reagents/single-color-antibodies-ruo/purified-mouse-anti-brdu.555627

Rabbit anti-Histone H1.2 (Proteintech, 19649-1-AP)
Species reactivity: H, M, R,
Application validated by manufacturer: WB, IP, IH, IF
https://www.ptglab.co.jp/products/HIST1H1C-Antibody-19649-1-AP.htm

Mouse histone H4 (clone:L64C1, Cell signaling, #2935)
Species reactivity: H, M, R, Mk
Application validated by manufacturer: WB, IH
https://www.cellsignal.jp/products/primary-antibodies/histone-h4-l64c1-mouse-mab/2935

Rabbit anti-MMP-9 (clone:AB19016, Millipore, #AB19016)
Species reactivity: H, M, R
Application validated by manufacturer: IH, IP, WB
https://www.merckmillipore.com/JP/ja/product/Anti-MMP-9-Antibody-Catalytic-domain,MM_NF-AB19016

Eukaryotic cell lines

Policy information about cell lines

Cell line source(s)	J774A.1 was obtained from JCRB Cell Bank. RAW264.7 was obtained from ATCC.
Authentication	None of the cell lines were authenticated.
Mycoplasma contamination	None of the cell lines were tested.
Commonly misidentified lines (See ICLAC register)	No commonly misidentified lines were used.

Animals and other organisms

Policy information about studies involving animals; ARRIVE guidelines recommended for reporting animal research

Laboratory animals	Non-transgenic C57BL/6J male mice (8-12 week-old) were used in this study.
Wild animals	No wild animals have been used for this study.

Field-collected samples	No field collected samples have been used for this study.
Ethics oversight	All experimental procedures were conducted following protocols approved by the Institutional Animal Care and Use Committee at the University of Tokyo (Permission No. P19-020).

Note that full information on the approval of the study protocol must also be provided in the manuscript.

Flow Cytometry

Plots

Confirm that:

☒ The axis labels state the marker and fluorochrome used (e.g. CD4-FITC).

☒ The axis scales are clearly visible. Include numbers along axes only for bottom left plot of group (a 'group' is an analysis of identical markers).

☒ All plots are contour plots with outliers or pseudocolor plots.

☒ A numerical value for number of cells or percentage (with statistics) is provided.

Methodology

Sample preparation	Mice were intraperitoneally administrated with 500 µl of a 4% TG solution (DIFCO Laboratories). After 72 h or the indicated time periods, ice-cold PBS (4 ml) was injected into the peritoneal cavity and 2.5 ml of lavage was collected. The peritoneal cells were washed once with ice-cold PBS and subjected to a flow cytometric analysis. For isolation of the peritoneal macrophages, peritoneal exudate cells collected 72 h after the TG injection were incubated in RPMI-1640 for 2 h at 37°C, wash twice with PBS, and the adherent cells (peritoneal macrophages) were collected using an enzyme-free cell dissociation solution (Sigma).
Instrument	BD FACSVerse, BD FACSAriaII
Software	Data were collected with the BD FACSuite and FACSDiva software and analyzed in FlowJo.
Cell population abundance	Between 10,000 and 30,000 cells were acquired per sample, and the total population was analyzed.
Gating strategy	Cells were defined based on FSC-A and SSC-A profile; Singlets were based on FSC-H and FSC-W profile, followed by SSC-H and SSC-W profile; finally, set gates with indicated staining: Macrophages (F4/80hi), Monocytes (Gr-1+, F4/80+), Dendritic cells (CD11c+), Neutrophils (Gr-1hi, F4/80-), and Eosinophils (SSChi). Fig. 5c and Fig. S8 provide information for FACS gating strategies.

☒ Tick this box to confirm that a figure exemplifying the gating strategy is provided in the Supplementary Information.
